# Reinforcement Efficiency of Cellulose Microfibers for the Tensile Stiffness and Strength of Rigid Low-Density Polyurethane Foams

**DOI:** 10.3390/ma13122725

**Published:** 2020-06-15

**Authors:** Jānis Andersons, Mikelis Kirpluks, Ugis Cabulis

**Affiliations:** 1 Polymer Laboratory, Latvian State Institute of Wood Chemistry, 27 Dzerbenes St., LV-1006 Riga, Latvia; mkirpluks@gmail.com (M.K.); cabulis@edi.lv (U.C.); 2 Institute for Mechanics of Materials, University of Latvia, 3 Jelgavas St., LV-1004 Riga, Latvia

**Keywords:** polymer matrix composites, rigid polyurethane foams, microcrystalline cellulose fibers, tensile strength, Young’s modulus

## Abstract

Rigid low-density closed-cell polyurethane (PU) foams are widely used in both thermal insulation and structural applications. The sustainability of PU foam production can be increased by using bio-based components and fillers that ensure both enhanced mechanical properties and higher renewable material content. Such bio-based foams were produced using polyols derived from rapeseed oil and microcrystalline cellulose (MCC) fibers as filler. The effect of MCC fiber loading of up to 10 wt % on the morphology, tensile stiffness, and strength of foams has been evaluated. For estimation of the mechanical reinforcement efficiency of foams, a model allowing for the partial alignment of filler fibers in foam struts was developed and validated against test results. It is shown that although applying MCC fibers leads to modest gains in the mechanical properties of PU foams compared with cellulose nanocrystal reinforcement, it may provide a higher content of renewable material in the foams.

## 1. Introduction

Rigid polyurethane (PU) and polyisocyanurate (PIR) foams have found diverse applications in construction, transport, and appliance industries, where their remarkable functional and structural properties are exploited. With sustainability becoming increasingly important, PU production from bio-based feedstock is being pursued in order to increase the renewable material fraction in foams, and also various fillers are applied to achieve the necessary functional characteristics at a lower density of reinforced foams, thus consuming less of the PU polymer [1,2].

The strength and stiffness of PU foams are determined by foam morphology and the mechanical properties of the PU polymer. The latter, depending on the chemical composition, may exhibit great variation in mechanical response, ranging from a flexible to a rigid material. Several factors affect the properties of PU, such as the cross-link density of the polymer matrix, hard/soft segment ratio, isocyanate type, isocyanate index, aromaticity, the presence of dangling side chains, packing and the segmental motion of PU chains [3]. Apart from functionality of the polyol and the type of isocyanate, PU foam properties also depend on such components of foam formulation as blowing agents, surfactants, and catalysts [1]. A convenient way to increase the stiffness of PU is by increasing the cross-link density of polymer via selecting polyols/isocyanates with a higher functional group content per molecule. However, although higher cross-link density results in a larger Young’s modulus of the rigid PU foams, it also leads to a reduction in the tensile strength and elongation at break of foams [4]. The intermolecular interactions and higher packing of polymer chains usually improve the mechanical properties of PU elastomers and flexible PU foams, whereas the high degree of cross-linking in rigid PU foams hampers the intermolecular conformation of polymer chains. Furthermore, both the strength and stiffness of rigid PU foams can be increased by filling the polymer with micro/nano-size particles, such as nanoclays and carbon nanotubes [5,6], glass fibers [7,8], and carbon fibers [9].

Nano- and microcrystalline cellulose (MCC) fibers can provide efficient mechanical reinforcement not only in monolithic polymer composites, but also in polymer foams due to the advantageous mechanical properties of fibers [10], which are small enough (see e.g., [10,11]) to be incorporated into cell walls and struts without affecting adversely the foam morphology. An additional benefit of using cellulose reinforcement in biopolymers is an opportunity of increasing the bio-based content of composite materials [11,12]. Cellulose nanofibers possess higher aspect ratio, stiffness, and strength than fibers of microcrystalline cellulose [10], which ensures a greater increase of the mechanical properties of nanofiber-reinforced composite at the same volume fraction of well-dispersed fibers. However, the production of cellulose nanofibers is still associated with high energy consumption and global warming potential [13] (although not exceeding those of other carbon nanomaterials such as carbon nanotubes and graphene [14]), which motivates further studies in the reinforcement efficiency of cellulose microfibers. MCC fibers have been applied as fillers of rigid PU foams comprising soybean [15] and rapeseed oil-based polyols [16,17], and the mechanical response of foams in compression has been studied. A consistent increase of the compressive stiffness and strength of foams with fiber weight fraction varying up to 9 wt % was found [17], despite the low aspect ratio of MCC fibers.

Relatively little modeling activity has accompanied experimental studies of the mechanical response for micro- and nanocellulose-filled foams, apparently due to complex interaction of cellulose and PU polymer in foams. Along with the aspect ratio and adhesion, the mechanical reinforcement efficiency of anisometric filler particles is strongly affected by their orientation. Cell growth during the foaming process is known to significantly affect the filler alignment in foams. Specifically, biaxial stretching of the cell wall material during cell expansion causes the orientation of fibrous and plate-like fillers along the cell wall, as observed for carbon nanofibers [18] and clay nanoplatelets [19,20,21]. Such an effect is also predicted by models of fiber motion in the vicinity of growing cells [22,23]. For low-density PU foams, most of the polymer is concentrated in cell struts, and the anisometric fillers would be expected to align with the strut axis due to the stretching of the struts during foam expansion. Indications for such a preferable alignment can be discerned in PU foams filled by, e.g., short milled carbon fibers [9], MCC fibers [15,17], and nanocellulose [24]. Furthermore, the predominant alignment of cellulose nanocrystals parallel to the PU foam rise direction has been reported [25]. Models have been proposed for numerical simulation [26] and analytical prediction [27] of the filler orientation distribution due to stretching of the polymer during the foaming process, demonstrating the additional reinforcing effect imparted by filler alignment.

In the present work, the effect of MCC fiber filler on the tensile strength and stiffness of rigid low-density bio-based PU foams is studied experimentally, thus complementing studies of the response of composite foams in compression [15,16,17]. The reinforcement efficiency of MCC fibers is evaluated and compared with the literature data for reinforcement of rigid PU foams by cellulose nanofibers. Analytical models for the reinforcement efficiency of foam stiffness and strength reflecting the filler alignment in foam struts are developed and validated.

## 2. Materials and Methods 

### 2.1. Materials

For ease of reference and completeness, formulation of the neat bio-based foams [6] is briefly recapitulated below. The polyol system of foams incorporated both polyols derived from rapeseed oil (RO) by amidization with diethanolamine employing zinc acetate as a catalyst (70 pbw) and higher functional polyether polyols based on sorbitol Lupranol 3422 (30 pbw) purchased from BASF (Ludwigshafen, Germany). NIAX Silicone L6915LV surfactant (1.5 pbw) produced by Momentive Performance Materials (Leverkusen, Germany). tris-chloropropyl phosphate flame retardant (30 pbw) supplied by Albemarle (Charlotte, NC, USA), and Polycat 5 catalyst (1 pbw) from Air Products (Halfweg, Netherlands) were also added to the polyol system. As a blowing agent, a mix of water (1 pbw) and cyclo-pentane (12 pbw) was used. Polymeric diphenylmethane diisocyanate IsoPMDI 92140 (164 pbw) supplied by BASF (Ludwigshafen, Germany) was the isocyanate component.

Ultrafine cellulose fibers ARBOCEL UFC 100 from Rettenmaier (Rosenberg, Germany) with a density of 1200 kg/m³, average fiber length of 8 µm, and average diameter of 2 µm were applied as fillers.

### 2.2. Foam Production

The fillers were added in predetermined quantities (0%, 1%, 3%, 5%, 7%, and 10% of neat foam polymer weight) to the blend of polyols, surfactant, catalyst, and flame retardant and stirred by mechanical mixer at 200 rpm for 3 min. Subsequently, the blowing agent was added, thus completing the polyol system. The foams were produced by adding isocyanate component, mixing for 10 to 15 s, and pouring the mixture into plastic molds of dimensions 20 × 30 × 10 cm for free foaming. The polymerization reaction took place at room temperature and was completed within about 3 to 5 min. 

### 2.3. Foam Characterization 

Upon setting of the foams, foam blocks were removed from the molds and conditioned at room temperature for 24 h. Since a denser surface layer had formed in the blocks produced, it was cut off before making specimens for tests.

**Figure 1:**
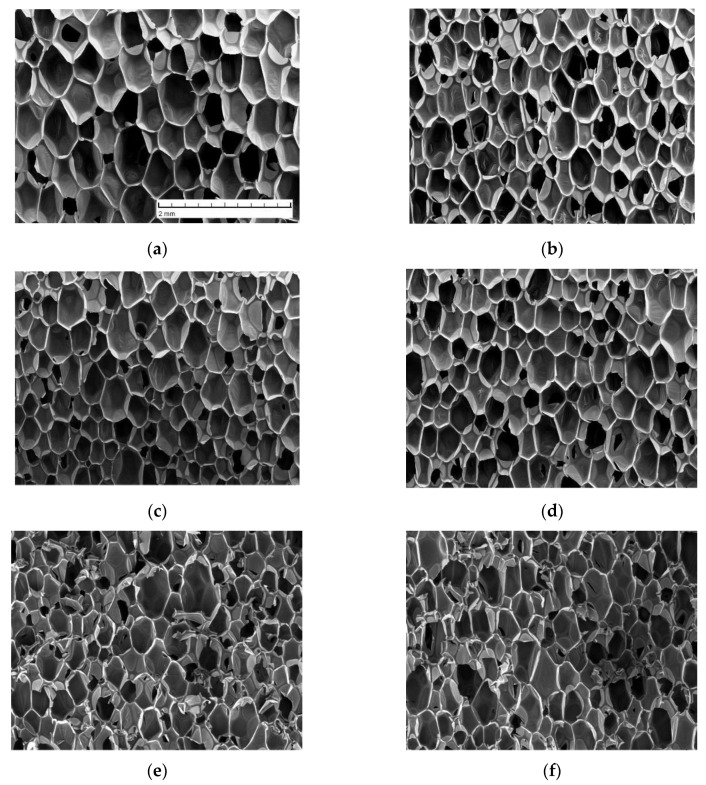
SEM images of foam cross-section in a plane aligned with the foam rise direction (the vertical direction in the pictures) at MCC fiber filler content of: (**a**) 0 wt % (neat foams); (**b**) 1 wt %; (**c**) 3 wt %; (**d**) 5 wt %; (**e**) 7 wt %; (**f**) 10 wt %.

Foam morphology was characterized by scanning electron microscopy (SEM). Foam samples of dimensions 1 × 1 × 0.2 cm were cut and sputtered with gold by using an Emitech K550X sputter coater (Emitech Ltd, Ashford, UK). Images of the surface of foam slices parallel to the foam rise direction were taken using a Tescan TS 5136 MM (TESCAN ORSAY HOLDING, a.s., Brno, Czech Republic, SEM) with a secondary electron detector; accelerating voltage was set to 15 kV, and the working distance was 15 mm. Representative micrographs of foams are presented in Figure 1. 

The images obtained were processed with a Vega TC software (version 2.9.9.21, TESCAN R&D Software Group, Brno, Czech Republic), and cell dimensions in the foam rise (cell length) and transverse (cell width) directions were measured. The average values and standard deviations of cell dimensions, as well as cell length and width ratio *R* characterizing geometrical anisotropy of foam cells, are listed in Table 1.

For mechanical tests, foam blocks were cut into slices along the foam rise direction, and the apparent density of each of the foam slices was determined. Specimens of dog-bone shape with rectangular test sections of 85 mm length, 22 mm width, and 20 mm thickness were machined from the foam slices for tests in the direction transverse to the foam rise.

Tensile tests were performed in stroke control at a loading rate corresponding to 10%/min in the gauge section by a servo-hydraulic test machine with a 1 kN load cell. The strain in the loading direction was measured by an extensometer MTS 634.25F-24 (MTS Systems Corporation, Eden Prairie, MN, USA) with a 50 mm gauge length.

## 3. Model

### 3.1. Mechanical Reinforcement Efficiency of Foams

The mechanical properties of polymer foams are determined by the foam porosity, anisotropy of foam cells, and the mechanical properties of the monolithic solid material forming cell walls and struts [28]. The introduction of reinforcing particles is primarily aimed at modifying foam characteristics via improving the properties of the solid polymer. However, the presence of filler can also affect the porosity and morphology of foams, e.g., due to the altering of viscosity of the foaming polymer or by filler particles acting as the bubble nucleation centers. Such changes in composite foam density and morphology also affect the mechanical properties of foams to an extent that can be commeasurable with mechanical reinforcement [29]. To separate the mechanical reinforcement effect from that of foam density variation, coefficients of modulus and strength enhancement defined as the ratio of the respective characteristics of composite and neat foams at the same apparent foam density have been introduced [30]. Furthermore, the degree of mechanical reinforcement for foam stiffness has been defined [31] so that the variation in cell shape (characterized by the shape anisotropy *R*) between neat and composite foams is also taken into account.

Mechanical reinforcement efficiency for stiffness *Γ_E_* is defined as the ratio of composite foam modulus *E_cf_* and neat foam modulus *E_f_*, the latter corresponding to the density *ρ_cf_* and geometrical anisotropy *R_cf_* of composite foams [31]:

(1) $$\Gamma_{E}=\frac{E_{cf}}{E_{f}\left(\rho_{cf},R_{cf}\right)}.$$

The neat foam stiffness can be expressed by a power function of foam density *ρ_f_* [28,31],

(2) $$E_{f}=c_{E}E_{s}{\left(\frac{\rho_{f}}{\rho_{s}}\right)}^{n_{E}}f_{E}\left(R\right)$$

where *E_s_* and *ρ_s_* designate the stiffness and density of the solid monolithic foam strut and wall material, *f_E_*(*R*) is a function reflecting the effect of cell shape anisotropy on foam stiffness, and *c_E_*, *n_E_* are constants to be determined via modeling [28] or by approximating the experimental data by Equation (2) [31]. Assuming that the dependence of stiffness of composite foams on their apparent density and geometrical anisotropy can be described by the same relation, Equation (2), upon substitution of neat solid material characteristics by the modulus *E_cs_* and density *ρ_cs_* of solid composite cell strut material:

(3) $$E_{cf}=c_{E}E_{cs}{\left(\frac{\rho_{cf}}{\rho_{cs}}\right)}^{n_{E}}f_{E}\left(R_{cf}\right)$$

and inserting foam stiffness expressions Equations (2) and (3) into Equation (1), the following relation for *Γ_E_* is obtained:

(4) $$\Gamma_{E}=\frac{E_{cs}}{E_{s}}{\left(\frac{\rho_{s}}{\rho_{cs}}\right)}^{n_{E}}$$

Further, expressing neat and composite monolithic material moduli *E_s_* and *E_cs_* from Equations (2) and (3), respectively, and substituting into Equation (4), the mechanical reinforcement efficiency for foam stiffness is derived in terms of foam characteristics as follows:

(5) $$\Gamma_{E}=\frac{E_{cf}}{E_{f}}{\left(\frac{\rho_{f}}{\rho_{cf}}\right)}^{n_{E}}\frac{f_{E}\left(R\right)}{f_{E}\left(R_{cf}\right)}\;\;.$$

Similarly, the mechanical reinforcement efficiency factor for foam strength *Γ_σ_* can be defined as the ratio of composite, *σ_cf_*, and neat foam strength, *σ_f_*, with neat foams having the same density and geometric anisotropy as the composite foams:

(6) $$\Gamma_{\sigma }=\frac{\sigma_{cf}}{\sigma_{f}\left(\rho_{cf},R_{cf}\right)}.$$

For low-density foams exhibiting brittle fracture or failing by the appearance of plastic hinges in the struts, foam strength is proportional to strength *σ_s_* of the solid strut material [28]. The foam strength can be expressed as

(7) $$\sigma_{f}=c_{\sigma }\sigma_{s}{\left(\frac{\rho_{f}}{\rho_{s}}\right)}^{n_{\sigma }}f_{\sigma }\left(R\right)$$

where *c_σ_*, *n_σ_* are foam morphology-related constants, and function *f_σ_*(*R*) allows for geometrical anisotropy effect on foam strength [28]. Assuming as above that relation Equation (7), upon replacing the relevant neat polymer characteristics by those of the composite solid, holds also for composite foam strength, it follows that the strength reinforcement efficiency according to Equation (6) is given by

(8) $$\Gamma_{\sigma }=\frac{\sigma_{cs}}{\sigma_{s}}{\left(\frac{\rho_{s}}{\rho_{cs}}\right)}^{n_{\sigma }}$$

and alternatively, via foam properties:

(9) $$\Gamma_{\sigma }=\frac{\sigma_{cf}}{\sigma_{f}}{\left(\frac{\rho_{f}}{\rho_{cf}}\right)}^{n_{\sigma }}\frac{f_{\sigma }\left(R\right)}{f_{\sigma }\left(R_{cf}\right)}\;\;$$

The reinforcement efficiency factors Equations (5) and (9) enable the separation of the purely mechanical effect of reinforcing particles on foam stiffness and strength from that caused by alteration in foam density and morphology, whereas Equations (4) and (8) provide a link between foam reinforcement efficiency and the properties of the solid composite material forming cell struts.

### 3.2. Stiffness and Strength of Foam Struts

In low-density closed-cell PU foams, 80% to 96% of the polymer is concentrated in cell struts [32,33]; therefore, the effect of cell walls on the mechanical properties can be neglected for a close conservative estimate of foam stiffness and strength [34]. The mechanical characteristics of such foams are determined by the axial stiffness and strength of foam struts via Equations (2) and (7) [28]. Hence, the mechanical reinforcement efficiency of foams can be predicted by Equations (4) and (8) once the composite strut properties are known. The latter can be evaluated by elementary micromechanical models as described below.

#### 3.2.1. Young’s Modulus

Young’s modulus *E_cs_* of a fiber-reinforced composite strut can be related to the Young’s modulus of polymer matrix *E_s_*, axial modulus of the reinforcing fibers *E_A_*, and fiber volume fraction *ν_f_* by a rule of mixtures type of relationship:

(10) $$E_{cs}=\eta_{oE}\eta_{lE}E_{A}\nu_{f}+\left(1-\nu_{f}\right)E_{s}$$

where *η_oE_* designates the fiber orientation factor and *η_lE_* is the fiber length efficiency factor. The latter can be expressed by a shear-lag model via the aspect ratio (i.e., length-to-diameter ratio) *κ* of the reinforcing fibers and stress transfer rate between the fiber and matrix *β* as follows:

(11) $$\eta_{lE}=1-\frac{\tanh\left(\beta \kappa \right)}{\beta \kappa }\;.$$

A number of analytical expressions for the stress transfer rate of various complexity and accuracy have been derived; however, they are mostly applicable to composites of relatively high fiber volume content. Since filler loading is usually relatively low in polymer foams, we use the relation for *β* shown to be accurate even for vanishing fiber volume fraction [35]

(12) $$\beta^{2}=\frac{2}{E_{A}E_{s}}\frac{E_{A}\nu_{f}+E_{s}\nu_{m}}{\frac{\nu_{m}}{4G_{A}^{*}}-\frac{1}{2G_{s}}\left(\frac{\nu_{m}}{2}+1+\frac{\ln\left(\nu_{f}+\chi \right)}{\nu_{m}}+\frac{\nu_{m}}{rD}\right)}.$$

In Equation (12), $\nu_{m}$. stands for the matrix volume fraction, $\nu_{m}=1-\nu_{f}$, $G_{A}^{*}=G_{A}/\left(1+2G_{A}/rD\right)$ is the effective fiber shear modulus, *G_A_* and *r* are fiber shear modulus and radius, respectively, *G_s_* denotes the shear modulus of the matrix, *D* designates a stiffness parameter of the fiber/matrix interface, and χ=0.009. is a numerically determined constant ensuring the accuracy of the expression for *β* at low fiber volume fractions [35]. 

If the distribution density p(θ). of the fiber orientation angle *θ*, i.e., the angle between fiber and strut axes, is known, fiber orientation factor $\eta_{oE}$. can be evaluated according to Krenchel’s approach (see e.g., [36]):

(13) $$\eta_{oE}=\int_{0}^{\pi /2}p\left(\theta \right)\cos^{4}\theta \sin\theta \;d\theta \;.$$

#### 3.2.2. Strength

For the axial tensile strength *σ_cs_* of a composite foam strut, the Fukuda and Chou model [37] in its modified form [38] is applied, expressing *σ_cs_* as a weighted sum of fiber axial strength *σ_A_* and matrix strength *σ_s_*:

(14) $$\sigma_{cs}=\eta_{o\sigma }\eta_{l\sigma }\nu_{f}\sigma_{A}+\left(1-\nu_{f}\right)\sigma_{s}$$

and employing fiber orientation, *η_o__σ_*, and length, *η_l__σ_*, efficiency factors for strength. The latter is presented in terms of reinforcing fiber length *l* and critical length *l_c_* as

(15) $$\eta_{l\sigma }=\{\begin{array}{cc} l/2l_{c} & l\leq l_{c}\\ 1-l_{c}/2l & l>l_{c} \end{array}.\;$$

where

(16) $$l_{c}=\sigma_{A}\;r/\tau$$

and *τ* stands for the interfacial shear strength (IFSS). Assuming a vanishingly small width of the critical zone, the fiber orientation factor *η_o__σ_* is also given by Equation (13) [38].

#### 3.2.3. Fiber Orientation Distribution

Upon vigorous mixing of the PU foam components, the orientations of filling fibers in the mixture are likely to be random, with uniform spatial and orientation distribution. During foaming, bubble nucleation and growth causes stretching of the struts, imparting preferential orientation of the fibers along the strut axis. We assume that the initial orientation distribution of filler is uniform, each strut undergoes isochoric stretching along its axis, and the anisometric filler particles are subjected to an affine rotation due to this stretching [27]. Then, the resulting fiber orientation distribution is symmetric about the strut axis, and the distribution density of the fiber orientation angle *θ* is given by (see, e.g., [39]):

(17) $$p\left(\theta \right)=\frac{\lambda^{3}}{{\left[\lambda^{3}+\left(1-\lambda^{3}\right)\cos^{2}\theta \right]}^{3/2}}$$

where *λ* is the stretch ratio of a strut, i.e., the degree to which the strut has been stretched during foaming. Fiber orientation factor $\eta_{o}=\eta_{oE}=\eta_{o\sigma }$ is obtained upon the substitution of p(θ) into Equation (13) and integration:

(18) $$\eta_{o}=\frac{\lambda^{3}}{2}\left(\frac{1+2\lambda^{3}}{{\left(\lambda^{3}-1\right)}^{2}}-\frac{3\lambda^{3}}{{\left(\lambda^{3}-1\right)}^{5/2}}{sec}^{-1}\lambda^{3/2}\right).$$

Since stretching of the structural elements of foams results from foam expansion, the stretch ratio should be a function of the foam expansion ratio $V_{foams}/V_{solid}$, i.e., the relative change in material volume during foaming; the expansion ratio is the inverse of the relative density of foams *γ*, $V_{foams}/V_{solid}=\rho_{s}/\rho_{f}=1/\gamma$. Assuming that the stretch ratio λ is the same for all the foam struts regardless of their length and orientation, it can be approximately related to the foam expansion ratio so that $\lambda^{3}=V_{foams}/V_{solid}=1/\gamma$, from which

(19) $$\lambda =1/\sqrt[{3}]{\gamma }\;.$$

## 4. Results and Discussion

As can be discerned in Figure 1 and Table 1, the presence of MCC filler caused a reduction in cell size by approximately 30%, which suggests that cellulose fibers acted as a nucleation agent facilitating the formation of bubbles during foaming. By contrast, the shape anisotropy of foam cells remained almost constant, exhibiting a very slight reduction with increasing fiber loading. The renewable material content of neat foams amounted to ca. 20 wt %. Incorporation of the cellulose filler increased the sustainable material fraction in composite foams up to about 28 wt % at the highest filler loading considered.

The MCC fibers had a positive effect on the tensile strength and stiffness of the foams, as seen in Table 2. Specifically, at 10 wt % fiber loading, the Young’s modulus of composite foams exceeded that of neat ones by about 45%, while the increase of strength amounted to ca. 17%. However, the larger fiber content was accompanied also by a growing apparent density of foams, presumably due to an increase in the viscosity of the polyol premix [17].

**Figure 2:**
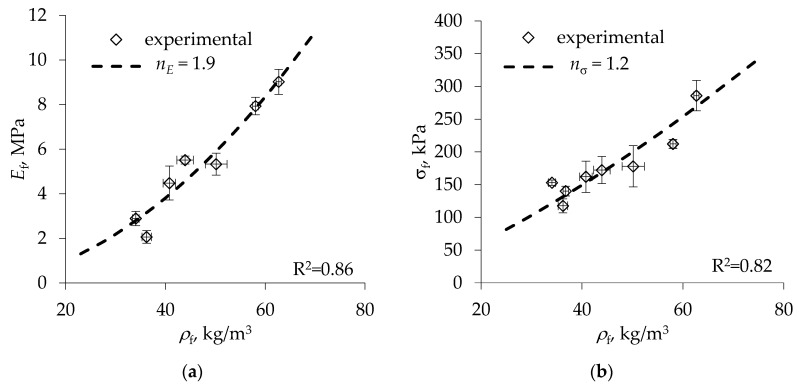
Variation of neat foam (**a**) modulus and (**b**) strength under tension in the transverse direction with foam density [34] and approximations of the data by Equations (2) and (7), as shown by dashed lines.

In order to estimate the mechanical reinforcement efficiency of foams by Equations (5) and (9), exponents *n_E_*, *n*_σ_ and shape anisotropy functions $f_{E}$, $f_{\sigma }$ entering the respective expressions need to be specified. For stiffness and strength in the direction transverse to the foam rise, the latter can be expressed in the form $f_{E}\left(R\right)=\left(R+1/R^{2}\right)/{\left(R+2\right)}^{2}$ and $f_{\sigma }\left(R\right)=\left(R+1\right){\left(R/\left(R+2\right)\right)}^{3/2}/R^{2}$ respectively by the rectangular-cell model; see [28,34]. As concerns the exponents of the density dependence of foam characteristics, we evaluated them by fitting Equations (2) and (7) to the experimental data for neat foams of the same formulation reported in [34]. The respective data are shown in Figure 2 together with best-fit approximations corresponding to *n_E_* = 1.9 and *n*_σ_ = 1.2. Notably, the value of the power-law exponent for the density dependence of foam stiffness is very close to the one derived for low-density open-cell foams and amounting to *n_E_* = 2, while the exponent for strength is lower than the predicted *n*_σ_ = 1.5 [28].

**Figure 3:**
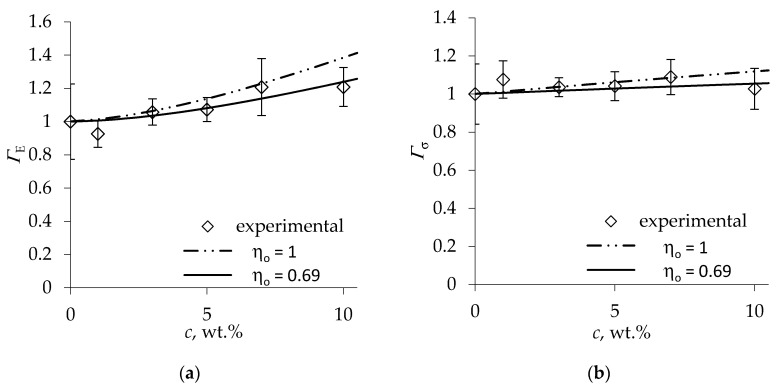
Mechanical reinforcement efficiency factors of composite foam (**a**) stiffness and (**b**) strength versus MCC fiber loading.

Mechanical reinforcement efficiency factors, evaluated by Equations (5) and (9) using the parameter values and the experimental data of Table 2, are presented in Figure 3 as functions of MCC fiber loading. It is seen that the correction for foam density and shape anisotropy has revealed the maximum reinforcement efficiency for stiffness of about 20% at 7 and 10 wt % fiber loading, the rest of the apparent gain in stiffness reflected in Table 2 being caused primarily by an increase in foam density due to the presence of the filler. The strength reinforcement efficiency, Figure 3b, is also markedly smaller than the apparent gain in strength suggested by the data in Table 2.

For theoretical estimation of the mechanical reinforcement efficiency factor of foam stiffness according to Equation (4), we rely on the MCC fiber properties reported in the literature. The axial modulus $E_{A}$. of deagglomerated, rod-like MCC fibers was evaluated at 25 GPa [40], and shear modulus $G_{A}$. of MCC at vanishing porosity was estimated as 3.5 GPa [41]. Due to good adhesion expected between the cellulose and PU polymer, the fiber/matrix interface stiffness parameter D→∞. implying perfect interface [35] is used in Equation (12). The stiffness and density of the neat polymer amounted to *E_s_* = 2.3 GPa and *ρ_s_* = 1210 kg/m^3^ [34]. The fiber volume fraction $\nu_{f}$ in the solid polymer is expressed via fiber weight fraction *c* (of neat polymer weight) as $\nu_{f}=c/\left(c+\varrho_{MCC}/\varrho_{s}\right)$ with $\varrho_{MCC}$ denoting the density of MCC fibers. Since the foam expansion ratios, calculated using the foam density values of Table 2, exhibited little variation ranging between 33.5 and 36.4, the average expansion ratio was applied to evaluate the fiber orientation factor by Equations (18) and (19), yielding *η_o_* = 0.69. The resulting dependence of *Γ_E_* on MCC fiber loading according to Equation (4) is plotted in Figure 3a by a solid line. A good agreement of the theoretical prediction with the efficiency factor values derived from test results is seen.

Concerning the reinforcement efficiency for strength, hydrogen [42] or even covalent [43] bonding between the cellulose and PU matrix ensures good adhesion; hence, stress transfer of the fiber/matrix interface in shear is likely to be limited by yielding of the PU polymer. Then, the IFSS *τ* entering the expression of fiber length efficiency factor Equation (15) can be roughly approximated by the shear yield strength of the solid polymer [44]. According to the von Mises yield criterion, the yield strength in shear is related to the tensile yield strength $\sigma_{s}$ as $\tau =\sigma_{s}/\sqrt{3}$; for the neat solid polymer, $\sigma_{s}$ = 61.5 MPa [34]. In the absence of experimental data regarding the axial tensile strength of MCC fibers, we substituted wood fiber strength [10] for $\sigma_{A}$ into Equation (16) to estimate the lower-bound value of the critical length of MCC fiber and found that $l_{c}>l$. It then follows from Equations (14) and (15) that the composite strut strength $\sigma_{cs}=\eta_{o\sigma }\kappa \tau \nu_{f}+\left(1-\nu_{f}\right)\sigma_{s}$. Using this relation, the predicted mechanical reinforcement efficiency for strength according to Equation (8) is plotted in Figure 3b by the solid line. It is seen that *Γ_σ_* derived from foam tests and from composite strength estimates agree well and are both rather low - the predicted reinforcement efficiency factor for 10 wt % loading amounts to 1.06, while the experimental *Γ_σ_* values are ca. 1.09 and 1.03 for 7 wt % and 10 wt % fiber content, respectively. For comparison, the predicted reinforcement efficiency for *η_o_* = 1 is also displayed in Figure 3, showing the level of *Γ_E_*, *Γ_σ_* attainable at a perfect fiber alignment with the strut axis.

To quantitatively evaluate the accuracy of prediction of the mechanical reinforcement efficiency factor for stiffness, the relative root mean square (RMS) error is calculated as follows:

(20) $$D_{E}=\sqrt{\frac{1}{N}\overset{N}{\underset{j=1}{\sum }}{\left(\frac{\Gamma_{E,\;\;ex}\left(c_{j}\right)-\Gamma_{E,th}\left(c_{j}\right)}{\Gamma_{E,ex}\left(c_{j}\right)}\right)}^{2}}\cdot 100\%$$

where $\Gamma_{E,ex}\left(c_{j}\right)$ denotes the stiffness reinforcement efficiency factor determined from foam test data at a filler loading $c_{j}$ by Equation (5), $\Gamma_{E,th}\left(c_{j}\right)$ is the respective predicted value according to Equation (4), and *N* is the number of filler loading levels considered. For strength, the relative RMS error *D_σ_* is calculated in the same way upon the substitution of $\Gamma_{E}$ by *Γ*_σ_ in Equation (20). The results are presented in Table 3. It is seen that the relative RMS error is less than 5% for the MCC fiber-filled foams.

Considerably higher gains in the mechanical properties of rigid low-density PU foams have been reported when applying cellulose whiskers as the filler, see e.g., [42,46]. Sucrose- and glycerol-based polyols and polymeric diphenylmethane diisocyanate (MDI) were used to produce the foams [42]. Composite foams were obtained by applying cellulose whiskers, derived from softwood craft pulp by sulfuric acid hydrolysis, as a filler at loadings c= 0.25 wt %, 0.5 wt %, 0.75 wt %, and 1 wt % of the total weight of polyols and MDI. Considering the tensile properties, the highest gains were obtained at 1 wt % loading of nanocellulose and amounted to about 227% for modulus and 99% for strength [42]. Notably, foam density also increased by ca. 52%, from 53.2 kg/m^3^ for neat foams to 82 kg/m^3^ for foams with the highest whisker content.

**Figure 4:**
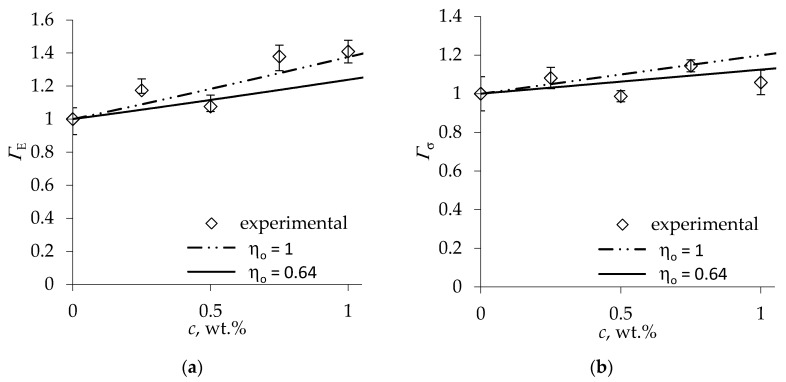
Mechanical reinforcement efficiency factors of composite foam (**a**) stiffness and (**b**) strength versus cellulose whisker [42] loading.

Since not only foam strength and stiffness but also density was markedly affected by the nanofiller, the density effect needs to be taken into account when evaluating reinforcement efficiency. In the absence of experimental data on density dependence of the neat foam stiffness and strength, we employed density exponent values *n_E_* = 2 and *n*_σ_ = 1.5 derived using rectangular unit cell model [28]. Since the presence of whiskers apparently affected cell size while no effect on the shape anisotropy of cells was reported in [42], *R_cf_* = *R* was assumed. The mechanical reinforcement efficiency factors evaluated by Equations (5) and (9) are presented in Figure 4. It is seen that the maximum gain in nanocomposite foam stiffness, when corrected for density variation, becomes ca. 40%, and for strength—14%.

For evaluation of the theoretical reinforcement efficiency factors of the nanocomposite, we employed the following estimates of filler properties: longitudinal whisker modulus $E_{A}$ = 151 GPa and strength $\sigma_{A}$ = 7.5 GPa [10], shear modulus $G_{A}$ = 15.5 GPa [47], and the aspect ratio of whiskers, produced by sulfuric acid hydrolysis of softwood craft pulp, *κ* = 50 [48]. The monolithic PU properties were assumed to be the same as quoted above. Since the total weight of polyols and MDI comprised 96 wt % of the neat PU foam formulation excluding the physical blowing agent [42], the volume fraction of nanocellulose in the PU polymer was expressed as $\nu_{f}=c\;/\left(c+\varrho_{cel}/0.96\varrho_{s}\right)$ where the nanocellulose density *ϱ_cel_* = 1600 kg/m^3^. Nanocomposite foams had slightly larger density than the MCC-filled ones; hence, the orientation factor calculated using the average expansion ratio of foams was smaller, amounting to *η_o_* = 0.64. The predicted efficiency factors *Γ_E_* and *Γ_σ_* of nanocomposite foams according to Equations (4) and (8) for *η_o_* = 0.64 are plotted in Figure 4 by solid lines. The modest increase in the reinforcement efficiency for strength with nanocellulose loading predicted via the strut strength model agrees very well with the *Γ_σ_* values derived from foam tests, as seen in Figure 4b, as also indicated by the respective relative RMS error of prediction shown in Table 3. However, the mechanical reinforcement efficiency of foam stiffness is closer to that predicted for fully aligned whiskers (*η_o_* = 1).

**Figure 5:**
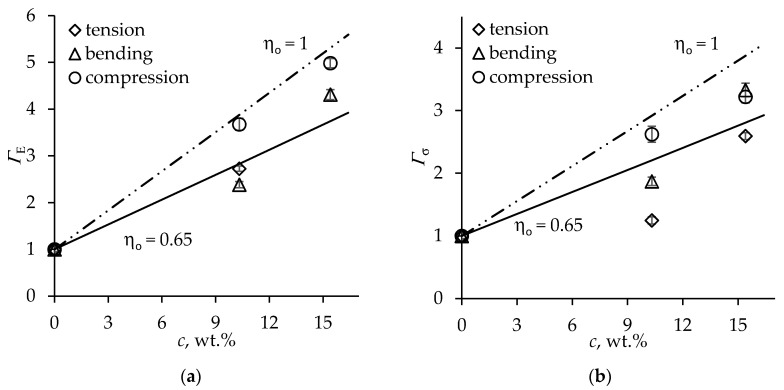
Mechanical reinforcement efficiency factors of composite foam (**a**) stiffness and (**b**) strength versus cellulose nanofibril [45] loading.

Considerably higher loadings of cellulose nanofibrils (CNF), 20 wt % and 30 wt % of the combined polyol and CNF weight, were applied for filling rigid low-density PU foams in [45]. Thus, the weight fraction of CNF in foams, according to the formulation presented in [45], amounted to 10.3 wt % and 15.4 wt %. A reduction in the cell size of composite foams but no effect on cell shape anisotropy was reported [45]; therefore, *R_cf_* = *R* was assumed when estimating the reinforcement efficiency factors by Equations (5) and (9). Substantial gains in the foam stiffness and strength in tension, bending, and compression were achieved, as shown in Figure 5. The variability of stiffness reinforcement efficiency among different loading modes seen in Figure 5a is likely to reflect the inter-batch variability of foams, the rate of growth of the bending and compressive stiffness with CNF loading being similar. However, the specific tensile stiffness at the highest filler loading was reported to increase by a factor of 10 [45], which appears to be inconsistent with the rest of data; hence, the respective data point was excluded from further analysis as an outlier. The reinforcement efficiency for strength, Figure 5b, exhibits marked scatter, tensile strength of foams benefiting from filler the least and compressive strength - the most. Such an effect is apparently caused by differing sensitivity in tension, bending, and compression to the stress concentrations caused by filler agglomerates in cell struts, defects in foam morphology, and superficial flaws introduced during the cutting of specimens.

For theoretical estimation of the reinforcement efficiency factors of CNF-filled foams, we employed the longitudinal modulus $E_{A}$ = 88 GPa determined for hardwood-derived CNF [49] and strength $\sigma_{A}$ = 1.6 GPa [50]. Due to the large aspect ratio of CNFs [10], the fiber length efficiency factor was taken as $\eta_{oE}=\eta_{l\sigma }=1$. The volume fraction of CNF in the PU polymer was expressed via the weight fraction as $\nu_{f}=c\;/\left(c+\left(1-c\right)\varrho_{cel}/\varrho_{s}\right)$. The properties of monolithic PU were assumed to be the same as above. The theoretical mechanical reinforcement efficiency of foams as a function of CNF loading according to Equations (4) and (8) is plotted in Figure 5 by solid lines for the partial alignment of fibers in the cell struts (*η_o_* = 0.65). It is seen that the predicted dependence of reinforcement efficiency on filler loading reasonably closely reflects the experimental trend, although the relative RMS error values are considerably larger, see Table 3, which is mainly due to the scatter among results for different loading modes. The predicted reinforcement efficiency for *η_o_* = 1, shown by broken lines in Figure 5, demonstrates the *Γ_E_*, *Γ_σ_* values theoretically attainable at a perfect CNF alignment.

Clearly, a much higher mechanical reinforcement efficiency of rigid PU foams can be achieved by the comparatively stiff, strong, and high aspect ratio cellulose whiskers and nanofibrils than by MCC fibers, even when corrected for variation in foam density and morphology, and for the selection of appropriate reinforcing filler for a given foam application, functionality, cost, and sustainability issues have to be balanced. The reinforcement efficiency factors for foam stiffness and strength can be applied as a tool in filler selection that reveals the intrinsic mechanical reinforcement effect of the filler.

## 5. Conclusions

MCC fibers have been considered as filler for enhancing the tensile stiffness and strength of rigid low-density bio-based PU foams, varying MCC loading up to 10 wt %. Foam stiffness increase by ca. 20% and strength - by 9% has been achieved. Theoretical estimates of the mechanical reinforcement efficiency factors for foam stiffness and strength are derived based on the rule-of-mixtures type of relations for the mechanical properties of struts of filled foams, taking into account the orientation of anisometric filler particles along the longitudinal direction of foam struts during foaming. The approach presented has been applied to the analysis of mechanical properties of low-density rigid PU foams reinforced by MCC fibers as well as by cellulose whiskers and nanofibrils. It is shown that reasonably close estimates of the mechanical reinforcement efficiency can be obtained by the proposed model.

## Figures and Tables

**Table 1:** Geometrical characteristics of foam cells as a function of microcrystalline cellulose fiber loading.

Fiber Loading, wt %	Cell Length, μm	Cell Width, μm	Shape Anisotropy *R*
0	745 (130) ^1^	480 (73)	1.56 (0.18)
1	567 (104)	364 (55)	1.56 (0.17)
3	514 (166)	345 (90)	1.48 (0.18)
5	565 (130)	362 (65)	1.56 (0.18)
7	502 (105)	333 (56)	1.51 (0.17)
10	498 (149)	337 (82)	1.47 (0.16)

^1^ Standard deviation is given in parentheses.

**Table 2:** Foam density, stiffness, and strength as a function of MCC fiber loading.

Fiber Loading, wt %	Foam Density, kg/m^3^	Young’s Modulus, MPa	Tensile Strength, kPa	Strain at Failure, %
0	33.1 (1.3) ^1^	3.36 (0.76)	127 (20)	6.6 (0.3)
1	33.0 (0.5)	3.10 (0.27)	137 (12)	7.6 (0.6)
3	34.3 (0.5)	3.90 (0.29)	142 (7)	6.4 (1.0))
5	33.4 (0.5)	3.66 (0.25)	134 (12)	5.9 (0.4)
7	35.6 (0.7)	4.74 (0.67)	152 (13)	5.6 (0.7)
10	35.8 (0.5)	4.86 (0.47)	148 (16)	5.0 (0.6)

^1^ Standard deviation is given in parentheses.

**Table 3:** Relative root mean square error of prediction of the mechanical reinforcement efficiency by cellulose micro- and nanofibers.

Relative RMS Error	Fibrous Cellulose Filler		
	MCC Fibers	Cellulose Whiskers [42]	Cellulose Nanofibrils [45]
*D*_E_, %	4.9	10.9	18.4
*D*_σ_, %	3.8	5.9	34.1
